# Single-nucleus transcriptome analysis of human brain immune response in patients with severe COVID-19

**DOI:** 10.1186/s13073-021-00933-8

**Published:** 2021-07-19

**Authors:** John F. Fullard, Hao-Chih Lee, Georgios Voloudakis, Shengbao Suo, Behnam Javidfar, Zhiping Shao, Cyril Peter, Wen Zhang, Shan Jiang, André Corvelo, Heather Wargnier, Emma Woodoff-Leith, Dushyant P. Purohit, Sadhna Ahuja, Nadejda M. Tsankova, Nathalie Jette, Gabriel E. Hoffman, Schahram Akbarian, Mary Fowkes, John F. Crary, Guo-Cheng Yuan, Panos Roussos

**Affiliations:** 1 Pamela Sklar Division of Psychiatric Genomics, Icahn School of Medicine at Mount Sinai, 1470 Madison Avenue, New York, NY 10029 USA; 2 Friedman Brain Institute, Icahn School of Medicine at Mount Sinai, 1470 Madison Avenue, New York, NY 10029 USA; 3 Icahn Institute for Data Science and Genomic Technology, Icahn School of Medicine at Mount Sinai, 1470 Madison Avenue, New York, NY 10029 USA; 4 Department of Genetics and Genomic Sciences, Icahn School of Medicine at Mount Sinai, 1470 Madison Avenue, New York, NY 10029 USA; 5 Department of Psychiatry, Icahn School of Medicine at Mount Sinai, 1470 Madison Avenue, New York, NY 10029 USA; 6 Dana-Farber Cancer Institute, Harvard Medical School, Boston, MA USA; 7 New York Genome Center, New York, NY USA; 8 Department of Pathology, Icahn School of Medicine at Mount Sinai, 1470 Madison Avenue, New York, NY 10029 USA; 9 Department of Neuroscience, Icahn School of Medicine at Mount Sinai, 1470 Madison Avenue, New York, NY 10029 USA; 10 Neuropathology Brain Bank & Research Core, Icahn School of Medicine at Mount Sinai, 1470 Madison Avenue, New York, NY 10029 USA; 11 Department of Neurology, Icahn School of Medicine at Mount Sinai, 1470 Madison Avenue, New York, NY 10029 USA; 12 Department of Population Health Science and Policy, Icahn School of Medicine at Mount Sinai, 1470 Madison Avenue, New York, NY 10029 USA; 13 Charles Bronfman Institute for Personalized Medicine, Icahn School of Medicine at Mount Sinai, 1470 Madison Avenue, New York, NY 10029 USA; 14 Mental Illness Research Education and Clinical Center (VISN 2 South), James J. Peters VA Medical Center, Bronx, 10468 NY USA

**Keywords:** Gene expression, Neuroinflammation, Prefrontal cortex, Choroid plexus, SARS-CoV-2, Microglia

## Abstract

**Background:**

Coronavirus disease 2019 (COVID-19), caused by severe acute respiratory syndrome coronavirus 2 (SARS-CoV-2) infection, has been associated with neurological and neuropsychiatric illness in many individuals. We sought to further our understanding of the relationship between brain tropism, neuro-inflammation, and host immune response in acute COVID-19 cases.

**Methods:**

Three brain regions (dorsolateral prefrontal cortex, medulla oblongata, and choroid plexus) from 5 patients with severe COVID-19 and 4 controls were examined. The presence of the virus was assessed by western blot against viral spike protein, as well as viral transcriptome analysis covering > 99% of SARS-CoV-2 genome and all potential serotypes. Droplet-based single-nucleus RNA sequencing (snRNA-seq) was performed in the same samples to examine the impact of COVID-19 on transcription in individual cells of the brain.

**Results:**

Quantification of viral spike S1 protein and viral transcripts did not detect SARS-CoV-2 in the postmortem brain tissue. However, analysis of 68,557 single-nucleus transcriptomes from three distinct regions of the brain identified an increased proportion of stromal cells, monocytes, and macrophages in the choroid plexus of COVID-19 patients. Furthermore, differential gene expression, pseudo-temporal trajectory, and gene regulatory network analyses revealed transcriptional changes in the cortical microglia associated with a range of biological processes, including cellular activation, mobility, and phagocytosis.

**Conclusions:**

Despite the absence of detectable SARS-CoV-2 in the brain at the time of death, the findings suggest significant and persistent neuroinflammation in patients with acute COVID-19.

**Supplementary Information:**

The online version contains supplementary material available at 10.1186/s13073-021-00933-8.

## Background

Coronavirus disease 2019 (COVID-19), caused by the novel severe acute respiratory syndrome coronavirus 2 (SARS-CoV-2), is currently the most urgent health care issue in the world. The central nervous system (CNS) is not the primary organ affected by SARS-CoV-2; however, there is increasing evidence that the brain is affected by SARS-CoV-2 by multiple mechanisms, each with potential short- and long-term impacts on neurological and cognitive function in affected individuals. As a result, SARS-CoV-2 has been associated with an unusually wide range of neurological and neuropsychiatric manifestations, ranging from asymptomatic cases to prolonged states of disability following initial recovery from COVID-19-associated delirium [1–4].

Systematically studying neurological disease in COVID-19 patients presents several challenges, including only a subset of the patient population displaying neurological symptoms, an inability to sample CNS tissues directly in living individuals, and difficulties in distinguishing direct neuro-invasion from systemic viremia within the brain. Brain autopsies and neuroimaging studies have demonstrated acute hypoxic and vascular injury [5, 6], as well as plausible SARS-CoV-2 CNS tropism [7–10]. Studies employing neuronal organoids have provided conflicting results regarding the effect of SARS-CoV-2 on neurons [9, 11, 12] or on epithelial cells in the choroid plexus [13, 14]. Overall, existing studies provide multiple mechanisms through which SARS-CoV-2 affects the human brain, including direct invasion and infection of specific types of neurons and glia, in addition to endothelial injury and vascular coagulopathy and diffuse neuroinflammatory processes or systemic inflammatory and hypercoagulable states.

To fully understand the impact of COVID-19 on the CNS, it is critical to perform a direct, unbiased, and comprehensive high-resolution exploration of the transcriptomic landscape in human brain tissue from patients with COVID-19. Here, we performed droplet-based single-nucleus transcriptome profiling in the brain regions that are potentially implicated in clinical manifestations of COVID-19: the prefrontal cortex, which is involved in higher-order cognitive function; medulla oblongata, which includes the respiratory center; and choroid plexus, which provides a toxin barrier to the brain and forms an interface between the blood and cerebrospinal fluid. The analysis described here examined the relationship between SARS-CoV-2 infection and neuroinflammation, brain tropism, and host immune response.

## Methods

### Samples used in this study

The study cohort consisted of tissue from 5 COVID-19 patients and 4 controls. For each donor, we studied 3 brain regions (PFC, medulla, and ChP). All specimens were obtained and de-identified by the biorepository at the Icahn School of Medicine at Mount Sinai in accordance with the policies and regulations at the Icahn School of Medicine at Mount Sinai (ISMMS) and its institutional review board. All samples were subjected to western blot, targeted RNA-seq, and RNA-FISH for viral spike protein, as well as transcriptomic analysis via snRNA-seq. We note that all COVID-19 cases were classified as severe; the clinical characteristics of donors are detailed in Additional file 1: Table S1.

### SARS-CoV-2 viral load quantification in frozen specimens of postmortem human brain

Brain tissue was harvested based on a modification of published protocols [15] for rapid dissection and systematic neuroanatomical sampling. For each donor, we targeted 3 brain regions, which included the dorsolateral prefrontal cortex (PFC), medulla oblongata (medulla), and choroid plexus (ChP). For the immunoblotting and SARS-CoV-2-targeted RNA-seq assay, we included a single dissection with the exception of the PFC, from which we included separate dissections of cortical gray matter and white matter. Western blot analysis was performed using 100 μg of total protein and anti-SARS-CoV-2 spike glycoprotein (Abcam, Cambridge, UK. Cat no: ab272504) or anti-β-actin (Abcam, Ab4970S) primary antibodies. For the SARS-CoV-2 targeted assay, we used the AmpliSeq Library Plus and cDNA Synthesis kits from Illumina (Illumina, San Diego, CA. Cat nos: 20019103 and 20022654), and, following quantification and pooling, libraries were run on a NovaSeq 6000 S4 (Illumina) in a 2 × 150 run format. For RNA-FISH, 10-μm sections were incubated with anti-sense DNA probes against the SARS-CoV-2 spike protein RNA sequence. Slides were incubated in TrueBlack Lipofuscin Autofluorescence Quencher (Biotium), and images were acquired on a Zeiss LSM780 confocal microscope. For further details, see Additional file 2: Supplementary methods.

### Immunohistochemistry

For immunohistochemical staining, fixed 40-μm sections of the choroid plexus from cases and controls were stained with anti-CD68 antibody (Abcam, ab955), and images were obtained on a Zeiss LSM780 confocal microscope and processed with ImageJ. For further details, see Additional file 2: Supplementary methods.

### Generation of single-nucleus RNA-seq from postmortem human brain

To better control for donor batch effects in the snRNA-seq analyses, we performed two dissections for each brain region and individual. The nuclei were extracted from 50 mg of frozen tissue. Each batch included 6 tissue samples, and the nuclei were incubated with individual nuclear hashing antibodies (BioLegend, San Diego, CA. TotalSeqA MAb414 anti-Nuclear pore complex protein) to facilitate multiplexed loading of the single-cell microfluidics device (10x Genomics, Pleasanton, CA). A total of 46,560 nuclei (7760 each) were loaded, in duplicate, on 10x Genomics B chips using 3′ capture chemistry. snRNA-seq and hash-tag oligo (HTO) libraries were generated separately and were sequenced by Nova-seq (Illumina) obtaining 2 × 100 paired-end reads.

### Single-nucleus data processing and demultiplexing

10x Genomics paired-end sequencing reads were processed and aligned on a pre-mRNA reference genome using cell ranger v3.1.0. HTO sequencing reads were preprocessed using kallisto indexing and tag extraction [16]. We customized an algorithm to identify singlets and doublets using HTO barcodes (see Additional file 2: Supplementary methods for more details). Demultiplexed unique molecular identifier matrices were combined and labeled by their associated experiment batch and brain region. The nuclei with fewer than 200 expressing genes or higher than 5% mitochondrial reads were omitted. We applied DoubletFinder [17] to remove doublets that were missed by HTO demultiplexing. We used Seurat [18] to perform data normalization, UMAP dimension reduction, and graph-based clustering. Harmony [19] was used to adjust batch effects using the first 50 principal components of the transformed count matrix. UMAP projection and Louvain clustering were then performed based on the shared nearest neighbor graph of these 50 harmonized principal components. We annotated clusters by inspecting a selected set of canonical markers.

Further clustering was performed on the pooled population of immune cells that includes microglia, monocytes, and macrophages. The clustering and UMAP visualization were based on 2000 variable genes of the pooled population. We applied Harmony [19] to calibrate batch effects. After manual inspection, a few clusters that were seemingly doublets were excluded from the subclustering analysis. We identified 9 clusters which are annotated as 7 subclusters of microglia (Mic-1 to Mic-7) and 2 subclusters of monocytes/macrophage (Mo-1 and Mo-2) based on the relative abundance of Mic and Mo/MP in each cluster. Marker genes of these clusters, comparing one cluster to the other 8 clusters, were calculated by the Wisconsin rank sum test as implemented in Seurat [18].

### Statistical analysis on cell composition

To identify cell composition changes associated with COVID-19, we computed the fraction of cells annotated with a cell type in each brain region and then fitted a linear mixed model:

$$ \mathrm{cell}\ \mathrm{fraction}\sim \mathrm{COVID}-19\mid \mathrm{tissue}+\left(1\ |\ \mathrm{tissue}\right)+\left(1\ |\ \mathrm{donor}\right). $$

Associated cell types were then identified by contrasting cases and controls for each tissue. We repeated statistical tests on the normalized cell fraction using centered log ratio transformation and obtained similar results.

### Identification of differentially expressed genes

Differentially expressed genes (DEGs) were identified using a linear mixed model. We first split the combined count matrix of 10,000 variable genes into sub-matrices containing the nuclei from one annotated cell population. *K*-nearest neighbor smoothing [20] was applied to each sub-matrix to impute dropped-out genes. Genes expressed in less than 1% of cells in a cell population were further filtered. A pseudo-bulk count matrix was formed by aggregating single-cell reads per sample. Using the dream pipeline [21], we fitted a mixed linear model to the pseudo-bulk count matrix:

$$ \mathrm{Log}\left(\mathrm{cpm}\left(\mathrm{Gene}\ \mathrm{expression}\right)\right)\sim \mathrm{COVID}-19\mid \mathrm{tissue}+\left(1\ |\ \mathrm{tissue}\right)+\left(1\ |\ \mathrm{donor}\right) $$

DEGs were then identified by contrasting cases and controls for each tissue. We omitted the test results on genes that were sparsely expressed (< 1% cells) in each tissue.

### Cell type enrichment

We performed Fisher’s exact test to compare gene signatures of annotated cell types with those reported in other studies. To generate the set of marker genes for an annotated cell type, we used the Wilcoxon rank sum test, implemented by the FindMarker function, to compare cells of one cell type against the rest. Up to 50 marker genes, with adjusted *P*-value less than 0.05 and ranked by fold changes, were included to form the set of marker genes. We curated gene sets of known cell types from 4 publicly available data sets [8, 22, 23], which are further described in Additional file 2: Supplementary methods. The background gene sets were set to be the intersection of 10,000 variable genes and all possible genes from the other data source.

### Construction and analysis of the transcription factor-gene network

We used pySENIC [24, 25] to identify transcription factor regulons. The count matrix of 10,000 variable genes was used. Genes expressed in less than 1% of cells were further filtered as recommended by the pySCENIC protocol. The gene co-expression network was inferred using the gradient boosting machine implemented by arboreto. Enriched motifs for a gene co-expression module were predicted using pre-computed databases from cisTargetDB and the ctx function in pySCENIC. Lastly, activity scores of inferred regulons were quantified at the single-cell level using AUCell.

To identify regulons associated with COVID-19, we first computed the averaged AUCell score per sample and fitted a linear mixed model:

$$ \mathrm{regulon}\ \mathrm{activity}\sim \mathrm{COVID}-19\mid \mathrm{tissue}+\left(1\ |\ \mathrm{tissue}\right)+\left(1\ |\ \mathrm{donor}\right). $$

Associated regulons were then identified by contrasting cases and controls for each tissue. We restricted our analysis to the 5 regulons that are most specific to a cell type ranked by the regulon-specific score [26].

### Pseudo-temporal trajectory score

To construct a pseudo-time trajectory that captures disease progression in the microglia, we computed the principal components using the gene count matrix of the top DEGs across three brain regions. We constructed a pseudo-time trajectory along the first two principal components using Slingshot [27]. The order of the pseudo time trajectory is chosen so that the fraction of cells from COVID patient is increasing with the pseudo-time. Gene expression profiles over the pseudo-time trajectory were analyzed using tradeSeq [28]. Applying resampling-based sequential ensemble clustering to genes with non-monotonic profiles, we identified 22 clusters, but a strong similarity between clusters was observed. Using a step-wise approach, we merged meta-clusters into 4 categories (see also Additional file 2: Supplementary methods).

### Transcriptome-wide association study and gene set enrichment

We used the “B2_ALL_eur_leave_23andme” summary statistics from the Release 4 (October 2020) of COVID-19 Host Genetics Initiative, which corresponds to the phenotype “Hospitalized covid vs. population, leave out 23andMe.” We employed blood and brain EpiXcan [29] tissue imputation models, trained in the STARNET [29, 30] and PsychENCODE [31, 32] cohorts, respectively. To derive gene-tissue-trait associations, transcriptomic imputation models were applied to the summary statistics following the S-PrediXcan approach [33]. For each tissue, we kept genes with *r*^2^ of the correlation between cross-validated prediction and observed expression greater than, or equal to, 0.01. For the gene set enrichment analysis, only protein-coding genes were considered, and the enrichment was tested with Fisher’s exact test.

## Results

### SARS-CoV-2 viral load quantification across multiple brain regions

**Fig. 1:**
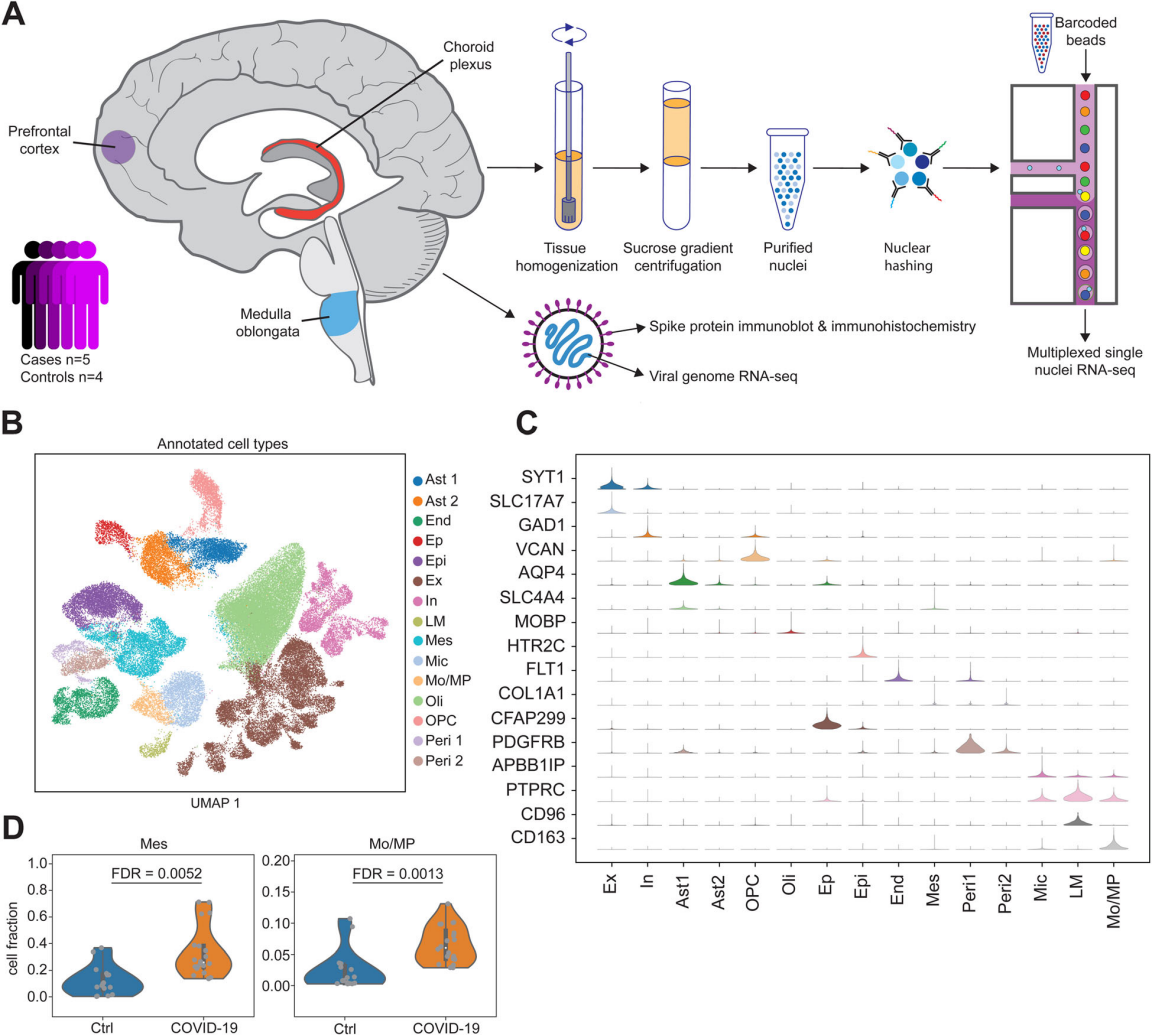
Droplet-based single-nucleus RNA sequencing in the dorsolateral prefrontal cortex (PFC), medulla oblongata (medulla), and choroid plexus (ChP) of 5 COVID-19 patients and 4 controls. **A** Experimental design. Frozen specimens of the human brain were dissected and subjected to a number of molecular assays, including single-nucleus RNA-sequencing (snRNA-seq), viral genome RNA-seq, and SARS-CoV-2 viral spike protein detection. **B** Uniform manifold approximation and projection visualization of annotated single-nucleus data (*n* = 68,557 barcodes). Colors show annotated cell types. **C** Distribution of canonical gene markers on annotated cell populations. The range of violins is adjusted by the maximum and minimum in each row. **D** Cell composition of mesenchymal cells and monocytes/macrophage in the choroid plexus stratified by case-control status. Only comparisons across tissues and cell types that survived false discovery rate correction are shown. Ast1 and Ast2 are the 2 groups of astrocytes. End, endothelial cells; Epi, epithelial cells; Ep, ependymal cells; Ex, excitatory neurons; In, inhibitory neurons; LM, lymphocytes; Mes, mesenchymal cells; Mic: microglia; Mo/MP, monocytes/macrophage; Oli, oligodendrocytes; Opc, oligodendrocyte progenitor cell. Peri1 and Peri2 are the two groups of pericytes

To characterize the CNS effect of SARS-CoV-2, we performed viral load quantification in human brain tissue from 5 COVID-19 patients and 4 controls (Fig. 1A). We note that all COVID-19 cases were classified as severe; the clinical characteristics of donors are detailed in Additional file 1: Table S1. For each donor, we targeted 3 brain regions (PFC, medulla, and ChP). Immunoblotting was negative for the presence of viral spike S1 protein in all tissues examined (Additional file 2: Fig S1). We then performed transcriptome analysis covering > 99% of SARS-CoV-2 genome and all potential serotypes. For each brain region and donor, we included a single dissection with the exception of the PFC, from which we included separate dissections of cortical gray matter and white matter (for an illustrative example, see Additional file 2: Fig S2), and generated, on average, 1.2 million reads per library. Across all samples, none of the sequencing reads mapped to the SARS-CoV-2 genome (Additional file 3: Table S2). In addition, examination of additional brain regions (red nucleus and substantia nigra), using fluorescence in situ hybridization (FISH) for SARS-COV-2 spike protein, also failed to detect the virus (Additional file 2: Fig S3). Overall, by employing three different experimental approaches, and exploring multiple brain regions, we did not detect SARS-CoV-2 in the postmortem brain tissue.

### snRNA-seq profiling across multiple brain regions in COVID-19 patients and controls

We characterized the molecular and cellular perturbations in the CNS of COVID-19 patients, independent of SARS-CoV-2 direct invasion, by performing droplet-based single-nucleus RNA sequencing (snRNA-seq) in the PFC, medulla, and ChP in the same set of 5 patients and 4 controls (Fig. 1A). To better control for donor batch effects, we performed two dissections for each brain region and individual (Additional file 2: Fig S2), and all samples per donor (*n* = 6) were pooled together using nuclear hashing. After preprocessing of snRNA-seq data, demultiplexing using hashtag-oligo intensity and quality control, 68,557 high-quality single-nucleus barcodes, with a median of 2817 detected genes per cell, were available for downstream analysis (Additional file 2: Fig S4). Variance in the gene expression was mostly driven by biological factors (cell type, brain regions, and donor) (Additional file 2: Fig S5).

We performed de novo taxonomy based on the graph-based clustering and uniform manifold approximation and projection (UMAP) across all brain regions and samples, and identified 15 major cell clusters (Fig. 1B). Clustering was independent of donor effect and technical variables, while differences between the brain regions were preserved (Additional file 2: Fig S6). Annotation of cell clusters based on the expression of canonical gene markers identified the following populations: excitatory neurons (Ex) that express *SYT1* and *SLC17A7*, inhibitory neurons (In) that express *SYT1* and *GAD1*, astrocytes (Ast1 and Ast2) that express *AQP4*, ependymal cells (Ep) that express *CFAP299*, oligodendrocyte progenitor cell (OPC) that express *VCAN*, oligodendrocytes (Oli) that express *MOBP*, epithelial cells (Epi) that express *HTR2C*, endothelial cells (End) that express *FLT1*, mesenchymal cells (Mes) that express *COL1A1*, pericytes (Per1 and Per2) that express *PDGFRB*, microglial cells (Mic) that express *APBB1IP*, and lymphocyte (LM) that express *CD96* and monocytes/macrophage (Mo/MP), expressing *CD163* (Fig. 1 and Additional file 2: Fig S7). Gene set enrichment analysis showed overlap for expected molecular pathways and functions, such as myelination for oligodendrocytes, chemical synaptic transmission for excitatory, and inhibitory neurons, and T cell activation for lymphocytes (Additional file 2: Fig S8). The expression profiles of each cell type show high concordance with previous snRNA-seq in human brain tissue [8, 22], peripheral blood cells, and brain organoids [13] (Additional file 2: Fig S9), indicating the robust definition of cell subpopulations in the current study.

### Compositional analysis identifies changes in the proportion of immune-related populations in COVID-19 patients

We assessed the relative proportions of the 15 major cell types in COVID-19 cases compared to controls across the three brain regions. For each cell cluster, we applied a linear mixed model to detect the interaction between COVID-19 cases and brain regions, while controlling for donor effects. Among 45 combinations of cell types and brain regions, we identified 2 cell populations from choroid plexus, including Mes cells and Mo/MP, showing a significant increase in their relative proportions in COVID-19 cases (Fig. 1D). We did not detect any significant COVID-19-associated changes in the cell type composition in either the PFC or medulla (Additional file 2: Fig S10). We next sought to confirm the presence of increased numbers of Mo/MP in the ChP by staining with an antibody against CD68, which is a marker for Mo/MP cells. We observe an abundance of CD68-positive cells in ChP of COVID-19 cases, and these cells do not appear to be associated with a blood vessel (Additional file 2: Fig S11). Overall, these results suggest that, in COVID-19, Mo/MP extravasate from the blood vessels into the stroma of the ChP, which is composed of Mes cells.

To further explore the cell states of immune-related cells associated with COVID-19 disease status, we then performed subclustering on the Mic and Mo/MP populations. We identified 9 clusters that corresponded to 7 subclusters of Mic and 2 subclusters of Mo/MP (Additional file 2: Fig S12a). The relative abundance of Mic-1 and Mic-2 subclusters in PFC showed significant changes in COVID-19 patients in the opposite direction compared to controls (Additional file 2: Fig S12b), suggesting a transition from Mic-1 to Mic-2 in PFC with COVID-19. Interestingly, expression of marker genes show that Mic-2 expresses, at a higher level, the Complement C3 gene (Additional file 2: Fig S12c) which has previously been shown to drive microglia activation [34]. The relative abundance of Mo/MP-1 in ChP increases significantly while Mo/MP-2 remains stable. Compared to Mo/MP-2, the Mo/MP-1 subcluster expresses LYVE1, which is a marker that is highly expressed in a subset of macrophages in the meninges [35].

### Differential gene expression analysis uncovered activation of innate immune cells in COVID-19 brain parenchyma

**Fig. 2:**
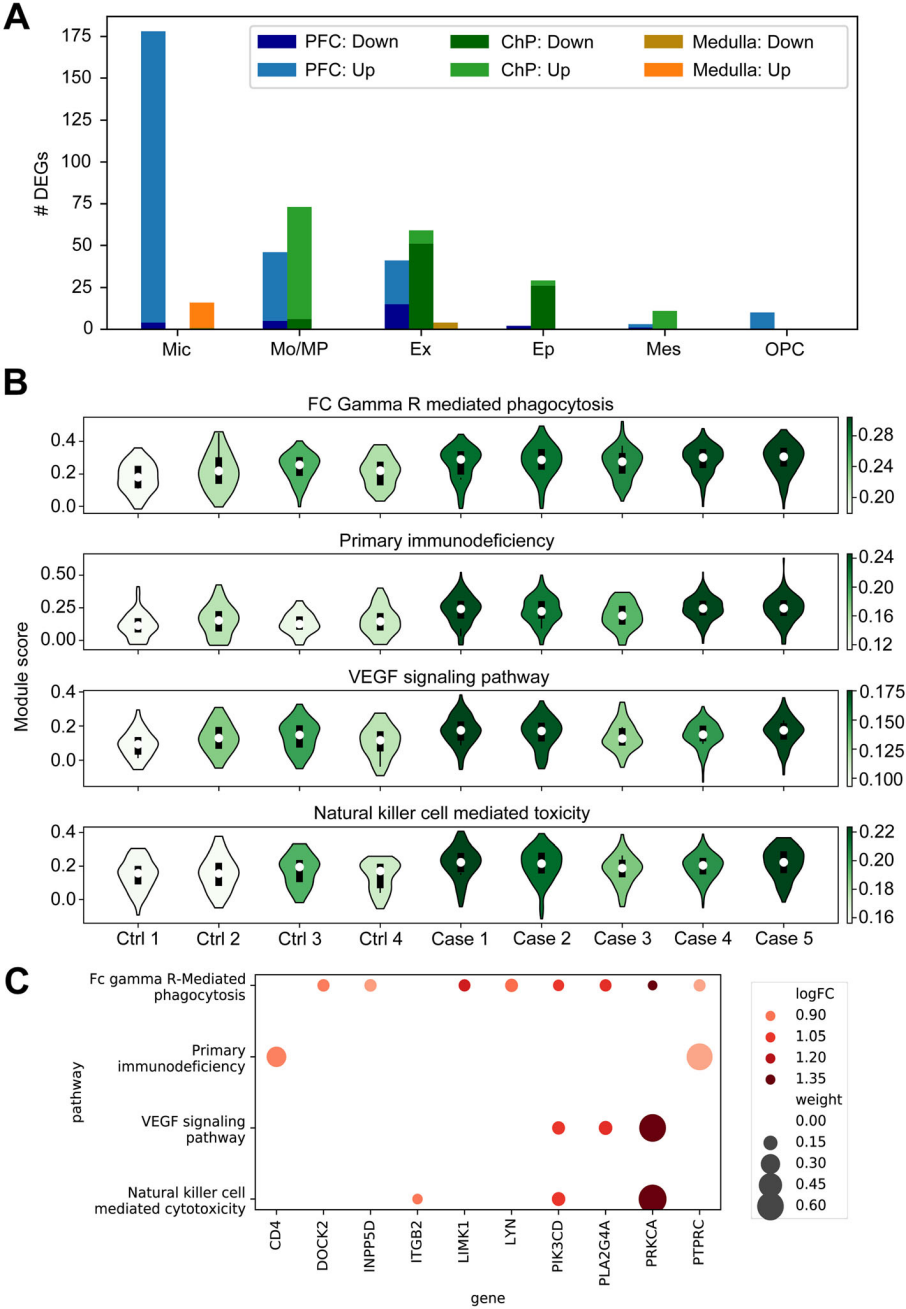
Differential gene expression and gene set enrichment analyses in COVID-19 patients compared to controls. **A** Number of differentially expressed genes (DEGs) identified in cell types across three brain regions. Up- and downregulated genes are shown in different colors. Cell types are ranked by the total number of DEGs across three brain regions. Cell types with no more than 10 DEGs in any brain region are omitted. **B** Gene set activity scores of PFC microglia. The four most significant pathways among 186 KEGG gene sets are shown. The shade of a violin indicates the median activity score of each individual. **C** Ten differentially expressed genes (FDR < 0.05) in four upregulated pathways are shown. The size of a circle shows the relative weight of a gene that contributed to the activity of a pathway. The relative weight is estimated as the ratio of the expression level of a gene to the sum of expression of all genes in the pathway. Colors show the log fold change

Molecular processes and biological pathways affected by disease states can be detected by studying the differences in gene expression among cases and controls within each cell subpopulation. For each cell type and brain region, we applied linear mixed models to identify differentially expressed genes (DEGs) among COVID-19 patients and controls, while controlling for donor effects. Among the 15 cell types and 3 brain regions, the microglia in the PFC showed the highest number of perturbations, including 178 DEGs (Fig. 2A), followed by monocytes in PFC and ChP. Interestingly, the majority of DEGs were upregulated in the immune cells (Mic and Mo/MP), indicating increased immune activity in COVID-19 patients. We provide a summary of differentially expressed genes across all cell types in Additional file 4: Table S3.

We examined whether gene perturbations affected specific biological processes. To preserve sufficient statistical power, we focused on the DEG signatures detected in microglia from the PFC. Gene set enrichment analysis identified biological processes such as “macrophage activation” (8 genes, *P* = 9.3 × 10^−6^) and “phagocytosis” (14 genes, *P* = 1.9 × 10^−6^) as being enriched with the 178 DEGs in PFC microglia (Additional file 5: Table S4). To further investigate transcriptomic changes in canonical pathways, we calculated the activity scores across 186 KEGG molecular pathways (Additional file 2: Fig S13). We then applied linear mixed models and identified differences in average activity levels across COVID-19 patients and controls. We identified 16 pathways showing significant differences in activity levels in the microglia of the PFC (Additional file 6: Table S5). As an illustrative example, we show the expression levels of the four most significant upregulated pathways (Fig. 2B) and associated genes (Fig. 2C). Interestingly, the majority of significant pathways were immune-related, and all of them, except one, which was related to “steroid biosynthesis,” were upregulated in COVID-19 patients. Depletion of steroid synthesis in COVID-19 patients is consistent with the clinical evidence that steroid administration is beneficial to modulate inflammatory response [36]. Overall, these results suggest the strong activation of innate immune cells in COVID-19 brain parenchyma.

### Pseudo-temporal analysis detected transition of inflammatory response in PFC across a range of biological processes

**Fig. 3:**
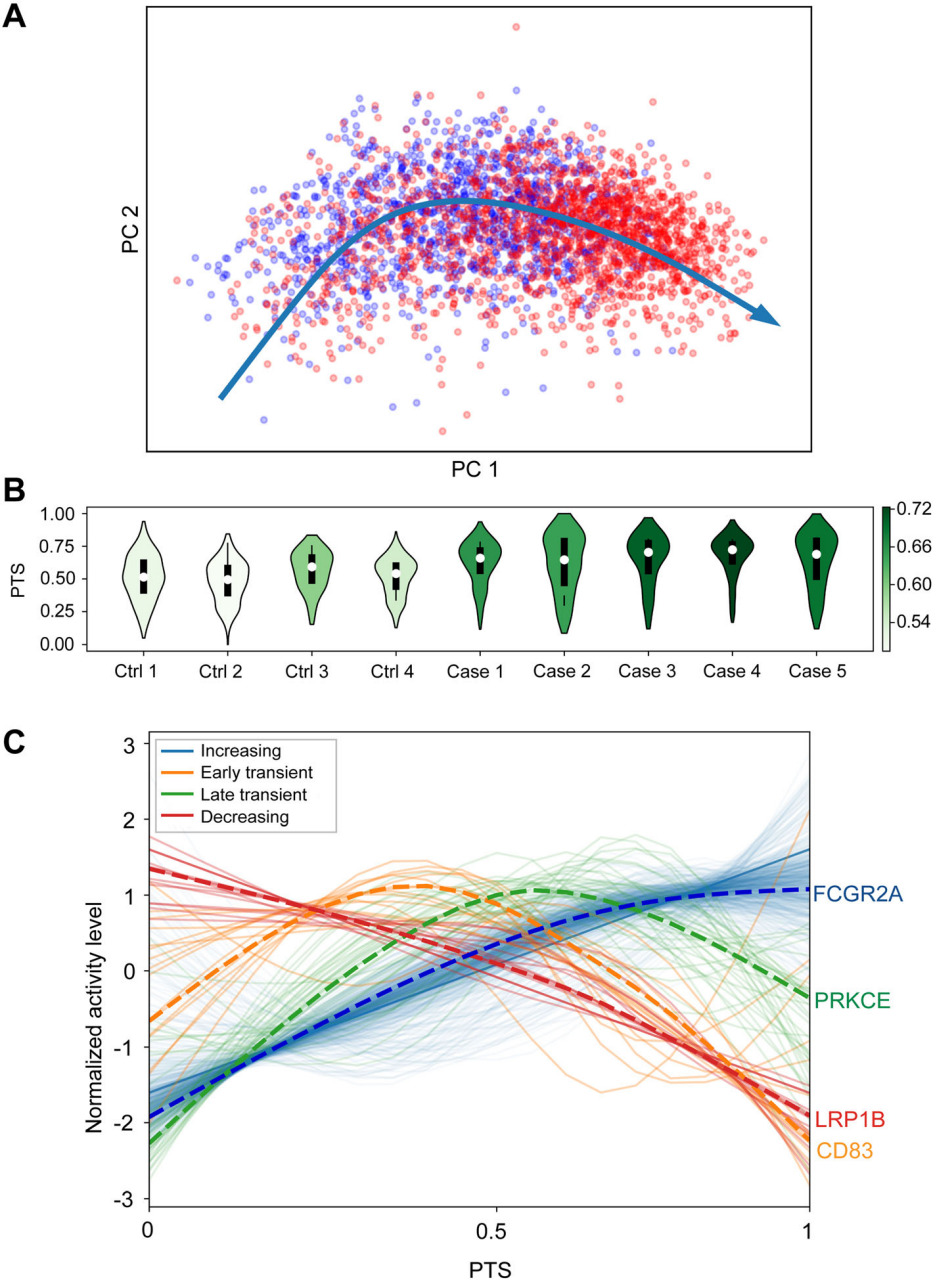
Pseudo-temporal trajectory score (PTS) analysis in the microglia-identified gene expression signatures with differential progression patterns in COVID-19 cases. **A** Pseudo-temporal trajectory in the microglia across three brain regions. Red and blue colors label cells from COVID-19 cases and controls, respectively. **B** PTS across 5 COVID-19 patients and 4 controls in PFC microglia. The shade of violin plots indicates the median activity score of each individual. **C** We identified 4 types of gene expression progression patterns over the pseudo-temporal trajectory: increasing (blue), early transient (orange), late transient (green), and decreasing (red). A dashed line shows the profile of a representative gene in each group

Statistical modeling can be applied to snRNA-seq to extract temporal information and study dynamic biological processes from cross-sectional data sets. We calculated a pseudo-temporal trajectory score (PTS) in the microglia, based on the progression of the transcriptional dysregulation in COVID-19 patients compared to controls (Fig. 3A). PTS has a wide distribution, potentially indicating microglia in different stages of activation and, as expected, COVID-19 patients demonstrated higher PTS than controls (Fig. 3B). To rule out patient-specific batch effects, we performed a similar analysis stratified by donor and did not observe any effect beyond case/control status (Additional file 2: Fig S14).

We categorized 646 commonly expressed microglia genes into four groups (increasing, decreasing, early transient, and late transient) based on the expression patterns capturing progressive changes related to PTS (Fig. 3C, Additional file 7: Table S6). The majority of genes were clustered as “increasing” (579 genes), followed by late transient (36 genes), early transient (16 genes), and decreasing (15 genes). Genes within the “increasing” cluster were more perturbed in COVID-19 patients (estimated based on *pi1* = 0.683), compared to the other 3 clusters (range of *pi1* = 0.051 to 0.077) and were enriched for 452 biological pathways, including “regulation of immune system process” (136 genes, *P* = 1.75 × 10^−16^) and “apoptotic process” (104 genes, *P* = 3.19 × 10^−5^). In the “early transient” group, 8 out of the 16 genes, including CD83 and 3 heat shock proteins (HSP90AA1, HSPB1, HSPH1), belong to the “cell activation” pathway (*P* = 6.40 × 10^−6^), while the “late transient” group was enriched for the “cell mobility” pathway (*P* = 4.44 × 10^−4^). Overall, the pseudo-temporal analysis supports a model where myeloid-driven inflammatory response in PFC involves transition across a range of biological processes, including cellular activation, mobility, and phagocytosis.

### Gene regulatory network (GRN) analysis identified activated microglia response in patients with COVID-19

Gene regulatory networks (GRNs) define the co-expression patterns of transcripts, considering the regulatory relationships between transcription factors (TFs) and their target genes. This analysis can determine cellular functions and model different systemic behaviors to uncover gene-level relationships in cells associated with disease states. To further understand the impact of SARS-CoV-2 on the TF-gene relationships, we explored the differences in GRNs among COVID-19 cases and controls. Across all cell subpopulations, we identified 131 TF modules that regulated, on average, 272 genes per module [25] (Additional file 8: Table S7). UMAP projection based on activity scores of GRNs reaffirmed the robustness of annotated cell types (Additional file 2: Fig S15).

**Fig. 4:**
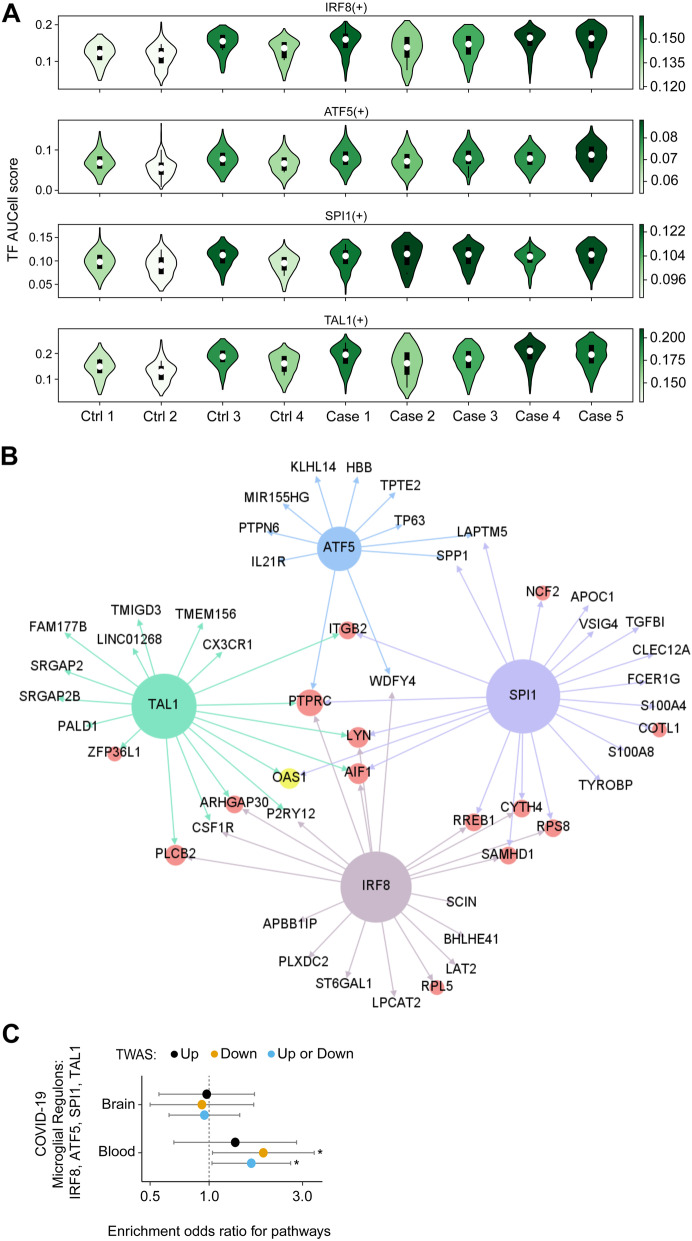
Gene regulatory network (GRN) analysis revealed transcription factors (TFs) driving transcriptomic dysregulation in COVID-19 patients. **A** TF module scores in PFC microglia. The shade of a violin indicates the median activity score of each individual. Four upregulated TF modules (IRF8, ATF5, SPI1, TAL1) are shown. **B** Upregulated TF modules in PFC microglia. Colored nodes show the transcription factors (blue, green, brown, and purple), DEGs (red), and a genetically associated gene based on genome-wide association studies (GWAS) (yellow). Nodes without circles are genes regulated by the transcription factors but are not DEGs. The regulatory network is trimmed to show only 14 DEGs, ranked by *P*-values, and 10 non-DEG genes regulated by each transcription factor. **C** Enrichment of the GWAS-associated genes in 4 microglia regulons: IRF8, ATF5, SPI1, and TAL1. Circles show odds ratios for the overlap of nominally significant GWAS gene (*n* = 285 and 560 for blood and brain, respectively, *P* ≤ 0.05), imputed from a GWAS comparing hospitalized COVID with respect to the general population, and genes of 4 microglia regulons. Error bars show 95% confidence intervals of estimated odds ratios. “Up” are those that are predicted to be upregulated (*n* = 140 and 297 for blood and brain, respectively, *P* ≤ 0.05) and “Down” are those that are predicted to be downregulated (*n* = 145 and 263 for blood and brain respectively, P ≤ 0.05). Analysis is limited to protein-coding genes only. Significant enrichments (*P* ≤ 0.05, Fisher’s exact test) are denoted by “*”

We defined cell population specificity by ranking TF modules according to regulon score [26] and uncovered well-known cell type-specific TFs, such as PAX6 for astrocytes and IRX8 for microglial cells (Additional file 2: Fig S16). We tested whether changes in the activity level of the top 5 TFs for each cell population were associated with COVID-19. Among the cell types and brain regions, TF modules in PFC microglia were the most affected (Additional file 2: Fig S17). We prioritized 4 out of 5 microglia PFC TFs (IRF8, ATF5, SPI1, TAL1) based on the upregulation in their activity in patients with COVID-19 (*Z* > 2.5, *P* < 0.05 and FDR within cell type < 0.05) (Fig. 4A; Additional file 9: Table S8). Projecting microglia DEGs onto the GRNs of these 4 TFs showed the co-regulatory TF-gene patterns affected in COVID-19 (Fig. 4B). Collectively, these results suggest GRNs corresponding to activated microglia response in patients with COVID-19 and nominate TFs that partially regulate those transcriptome changes.

### GRNs corresponding to microglia activation in acute COVID-19 patients demonstrate enrichment for genes that are predicted to be downregulated in a transcriptome-wide association study (TWAS) of severe COVID-19

To assess if acute microglia activation has a beneficial or deleterious effect, we examined whether the direction of effect of transcriptome signatures is concordant with genetically regulated changes in the gene expression that are associated with severe COVID-19. We leveraged genetic variation from the COVID-19 Host Genetics Initiative and the gene expression models from the brain [31, 32] and blood [30] tissues to impute the genetically regulated transcriptomic changes [29] associated with severe COVID19 outcomes (Additional file 10: Table S9). Since microglia gene expression models are not currently available, we included proxy tissue (homogenate brain and whole blood) models to capture the transcriptome profiling of the microglial cell lineage. Given that the microglia only represent 0.5 to 16.6% of all cells in the brain parenchyma [37], we also included blood tissue models corresponding to immune cells which are close to the microglial cell lineage [38, 39]. We identified 12 significant genes (at FDR 5%) in the brain and blood (*AP000295.1*, *CCR3*, *CR936218.2*, *CRHR1*, *FYCO1*, *IFNAR2*, *IL10RB*, *IL10RB-AS1*, *LRRC37A4P*, *MAPT-AS1*, *OAS1*, *OAS3*) that were associated with hospitalized COVID-19 patients, with respect to the general population. Nominally significant gene-trait associations (*P* < 0.05) from the imputed blood transcriptome were enriched (OR = 1.64, *P* = 0.030, Fisher’s exact test) with GRNs that are associated with the top 4 microglia TFs (IRF8, ATF5, SPI1, TAL1) (Fig. 4C). In a secondary analysis, we examined whether the genetic liability was associated with a predicted downregulation or upregulation of the genes participating in these TF modules. Enrichment was observed only for genes predicted to be downregulated (OR = 1.89, *P* = 0.035, Fisher’s exact test), compared to genes predicted to be upregulated in susceptible individuals (OR = 1.35, *P* = 0.25, Fisher’s exact test). In addition, *OAS1*, which was predicted to be downregulated in susceptible individuals (FDR-adjusted *P* = 0.003), was involved in 3 microglia GRNs (SPI1, IRF5, TAL1, Fig. 4B). Overall, one possible interpretation of these observations is that these TF modules, which are activated in the microglia of acute COVID-19 patients and are predicted to be hypo-active at baseline in susceptible individuals, may have a beneficial role in the host response.

## Discussion

Recent advances in single-cell approaches provide an opportunity to study highly complex tissues like the human brain at unprecedented resolution. In order to better understand the impact of acute COVID-19 on the CNS, we studied the effects of COVID-19 in individual cells across 3 functionally distinct regions of the human brain (prefrontal cortex, choroid plexus, and medulla oblongata). Although no virus was detected, single-nucleus gene expression analysis revealed extensive differences in the brains of COVID-19 patients when compared to controls; specifically in the ChP and PFC. We observed a relative increase in the proportions of infiltrating immune cells in the ChP, suggesting potential migration of monocytes/macrophages across the blood-brain barrier. Microglia residing in the PFC of COVID-19 patients displayed dysregulated gene expression. The majority of the microglial DEGs were upregulated, mediating a myeloid-driven inflammatory response that involved a range of biological processes, including cellular activation, mobility, and phagocytosis. This is consistent with previous studies that have also described increased inflammatory response of microglia in COVID-19 cases [8, 10]. Finally, by leveraging genetic variation to infer differences in COVID-19 susceptible individuals, we provided preliminary evidence for a potential beneficial role for microglia activation during the acute COVID-19 phase.

Although there is evidence that SARS-CoV-2 spike protein can be detected in the brain, including the cortex [9], choroid plexus [8], and medulla oblongata [10, 40], immunoblotting, immunohistochemistry, and viral genome RNA-seq indicate that the virus was not present at the time of death in the specimens included in this study. The ability to detect SARS-CoV-2 in the CNS is affected by the duration of COVID-19 infection, with a marked decrease in detectable virus by day 20 [10]. In our study, all individuals had been infected for ≥ 14 days while the duration of infection was ≥ 30 days for 3 out of 5 COVID-19 cases. In addition, only a subset of COVID-19 patients indicates non-zero SARS-CoV-2 RNA copies in the CNS, which are more difficult to detect in the brain parenchyma compared to the olfactory mucosa [10]. Although our focus was on immune cells, there is evidence, in addition to microglia activation, for COVID-19-related transcriptional changes in a range of brain cell types including astrocytes, oligodendrocytes, and excitatory neurons [8]. The observed differences in the number of DEGs, and the cell types affected, might be explained by the experimental design: two versus a single dissection per brain region and individual. If we only consider a single dissection per brain region and individual in our analysis, the number of DEGs increases (data not shown) and includes perturbations among every major CNS cell type.

## Conclusions

Taken together, these findings indicate persistent activation of the innate immune response in the brains of patients with COVID-19. Based on our results, it is possible that the inflammatory response of microglia is induced as a result of peripheral immune cells infiltrating CNS through the blood-brain barrier. Another point of entry of SARS-CoV-2 to the CNS is by crossing the neural-mucosal interface in olfactory mucosa [10]. These two mechanisms are non-mutually exclusive and might be associated with different stages of disease progression and presentation of clinical symptoms. While shedding light on the impact of SARS-CoV-2 on the CNS, our study was insufficiently powered to fully elucidate all of the relevant cellular states associated with COVID-19, and future research efforts are required to confirm, and expand on, our findings. In conclusion, our study suggests extensive neuro-inflammation and brain immune response in acute COVID-19 patients, even in the absence of direct evidence of SARS-CoV-2 neuro-invasion.

## Supplementary Information

**Additional file 1: Table S1.** Clinical characteristics of donors.

**Additional file 2: **Supplementary methods. Supplementary figures: **Fig S1.** Western Blot Analysis Investigating the presence of COVID-19 in Human Brain. **Fig S2.** Brain specimen dissections. **Fig S3.** SARS-COV2 RNA not detected in the postmortem midbrain of COVID-19 patients. **Fig S4.** Quality control of single nucleus data. **Fig S5.** Variance partition plot. **Fig S6.** Cell type composition across 15 major cell clusters. **Fig S7.** UMAP visualization of the distribution of canonical gene markers on annotated cell populations. **Fig S8.** Gene set enrichment analysis for marker genes of annotated populations. **Fig S9.** Comparison of marker genes of annotated populations from the current study and other published studies. **Fig S10.** Cell type composition among cases and controls. **Fig S11.** Immunohistochemistry of CD68 positive cells in the Choroid Plexus. **Fig S12.** Sub-clustering of microglial and monocyte/macrophage populations. **Fig S13.** KEGG pathway enrichment network. **Fig S14.** Pseudo-temporal trajectory score (PTS) analysis in microglia identifies gene expression signatures across different donors. **Fig S15.** UMAP projection based on the activity scores of 131 TF regulons. **Fig S16.** Regulon specificity scores (RSS) of each annotated cell population. **Fig S17.** Transcription factor activity scores across different cell types and brain regions.

**Additional file 3: Table S2.** Sequencing statistics for SARS-CoV-2 targeted RNA-seq.

**Additional file 4: Table S3.** Differentially expressed genes.

**Additional file 5: Table S4.** Gene Ontology term enrichment in differentially expressed genes of PFC microglia.

**Additional file 6: Table S5.** Differences in gene set activity scores among COVID-19 patients and controls.

**Additional file 7: Table S6.** Genes clustered by pseudo time analysis.

**Additional file 8: Table S7.** Transcription regulatory modules estimated by SCENIC.

**Additional file 9: Table S8.** Differences in TF regulon activity scores comparing COVID-19 patients and controls.

**Additional file 10: Table S9.** Imputed gene associations from COVID-19 GWAS.

## Data Availability

Processed and raw data can be downloaded from NCBI GEO (GSE164485) [41]: https://www.ncbi.nlm.nih.gov/geo/query/acc.cgi?acc=GSE164485. Scripts used in this study are available on GitHub [42]: https://github.com/howchihlee/covid_brain_sc.

## Abbreviations

COVID-19: Coronavirus disease 2019
SARS-CoV-2: Severe acute respiratory syndrome coronavirus 2
snRNA-seq: Single-nucleus RNA sequencing
FISH: Fluorescence in situ hybridization
CNS: Central nervous system
PFC: Prefrontal cortex
ChP: Choroid plexus
HTOs: Hash-tag oligos
DEGs: Differentially expressed genes
Ex: Excitatory neurons
In: Inhibitory neurons
Ast: Astrocytes
Ep: Ependymal cells
OPC: Oligodendrocyte progenitor cell
Oli: Oligodendrocytes
Epi: Epithelial cells
End: Endothelial cells
Mes: Mesenchymal cells
Per: Pericytes
Mic: Microglial cells
LM: Lymphocyte
Mo: Monocytes
MP: Macrophages
UMAP: Uniform manifold approximation and projection
PTS: pseudo-temporal trajectory score
HSP90AA1, HSPB1, HSPH1: Heat shock proteins
GRNs: Gene regulatory networks
TFs: Transcription factors
GWAS: Genome-wide association studies
